# Sustained-Release Solid Dispersion of High-Melting-Point and Insoluble Resveratrol Prepared through Hot Melt Extrusion to Improve Its Solubility and Bioavailability

**DOI:** 10.3390/molecules26164982

**Published:** 2021-08-17

**Authors:** Wenjing Zhu, Wenling Fan, Xiaotong Zhang, Meiqi Gao

**Affiliations:** 1 Laboratory of Pharmacy Engineering, College of Pharmacy, Nanjing University of Chinese Medicine, Nanjing 210023, China; zwj19970114@163.com (W.Z.); aixiaotong95@163.com (X.Z.); gmq3064@163.com (M.G.); 2 Jiangsu Collaborative Innovation Center of Chinese Medicinal Resources Industrialization, School of Pharmacy, Nanjing University of Chinese Medicine, Nanjing 210023, China

**Keywords:** solid dispersion, resveratrol, hot melt extrusion, dissolution, sustained-release

## Abstract

This study aimed to prepare a sustained-release solid dispersion of poorly water-soluble resveratrol (RES) with high melting point in a single hot melt extrusion step. A hydrophobic–hydrophilic polymeric blend (Eudragit RS and PEG6000) was used to control the release of RES. With the dispersive mixing and high shear forces of hot melt extrusion, the thermodynamic properties and dispersion of RES were changed to improve its solubility. The effects of the formulation were investigated through univariate analysis to optimize the preparation of the sustained-release solid dispersion. In vitro and in vivo studies were performed to evaluate the prepared RES/RS/PEG6000 sustained-release solid dispersion. The physical state of the solid dispersion was characterized using differential scanning calorimetry and X-ray diffraction. Surface properties of the dispersion were visualized using scanning electron microscopy, and the chemical interaction between RES and excipients was detected through Fourier-transform infrared spectroscopy. Results suggested that the optimized sustained-release solid dispersion was obtained when the mass ratio of RES-polymeric blend was 1:5, the ratio of PEG6000 was 35%, the barrel temperature was 170 °C, and the screw speed was 80 rpm. In vitro studies demonstrated that the solid dispersion showed a good sustained release effect. The cumulative release of RES reached 82.42% until 12 h and was fit by the Weibull model. In addition, the saturated solubility was 2.28 times higher than that of the bulk RES. In vitro studies demonstrated that the half-life increased from 3.78 to 7.09 h, and the bioavailability improved to 140.38%. The crystalline RES was transformed into the amorphous one, and RES was highly dispersed in the polymeric blend matrix.

## 1. Introduction

Traditional Chinese medicine (TCM) has many advantages, such as low toxicity, minimal side effects, and widespread use [1,2,3]. At present, oral administration is still the preferred method of TCM treatment. Many TCM active pharmaceutical ingredients, such as resveratrol (trans-3,5,4′-trihydroxystilbene, RES) and emodin, possess good curative effects for the treatment of cancer and cardiovascular diseases. Given their poor solubility [4,5,6], high-melting-point drugs are often difficult to dissolve in aqueous solvent systems because of their strong crystalline structure, greatly affecting their absorption and bioavailability [7,8]. RES is a natural substance possessing various biological activities, such as anti-inflammatory, antioxidant, cardioprotective, and anticancer [9]. However, the oral bioavailability (less than 5%) of RES is limited by its poor solubility, oxidation, and rapid metabolism [10]. Therefore, effective and suitable drug delivery systems (DDSs) for RES are needed to address the aforementioned drawbacks.

Over the last few decades, conventional dosage forms are being replaced by new DDSs that improve the therapeutic effectiveness of insoluble drugs. Solid dispersion (SD) technology is a common formulation strategy to increase solubility by changing the porosity, particle size, and wettability of poorly soluble drugs [11]. Compared with liposomes and nanoparticles, SDs have higher drug loading and stability. SDs can be prepared through several techniques, such as spray-drying, solvent evaporation, and hot-melt extrusion (HME) [12]. Among these techniques, HME is a solventless technique suitable for large-scale industrial production [13]. Its advantages include simple process, high degree of automation, continuous operation, high production efficiency, and on-line monitoring. It has a unique blending geometry, promotes mixing, and induces high shear forces to achieve uniform drug dispersion in the carrier [14].

Although HME is a promising method, it is limited by its inability to produce high-melting-point and heat-sensitive drugs [15]. Large segment polymers are used as sustained-release carriers, some of which possess a high glass transition temperature (Tg). At low temperatures, high-melting-point drugs and carriers cannot be completely melted and dispersed, and excessively high process temperature may lead to drug decomposition and degradation [16]. The physicochemical properties and phase state of the drug or the whole system can be changed through preprocessing methods, such as reducing drug particle size, decreasing melting point, and preparing amorphous drugs, to increase the application of HME in the preparation of high-melting-point drug SDs. Lakshman et al., used the solvent method to convert a drug with high melting point to an amorphous form before preparing an SD by HME [17], of which solvents present issues related to toxicity, environmental impact, and operating cost [18]. Other studies selected and added appropriate auxiliary materials, such as poloxamer 188, poloxamer 407, polysorbate 80, and d-alpha tocopheryl polyethylene glycol 1000 succinate [19,20,21], which facilitate the melting and mixing of drugs and carriers at low temperatures.

Sustained-release (SR) dosage forms can reduce the dosing frequency by maintaining therapeutically effective drug concentrations over a prolonged period of time and improve patient compliance. Among various formulation approaches for sustained drug release, preparing matrices with insoluble polymers using the SD technique is an effective way to produce SR oral dosage forms [22]. In polymer matrix systems, drug molecules are homogeneously distributed throughout a matrix, and drug release rate is controlled by water-soluble or erodible matrices consisting of various hydrophilic or hydrophobic polymeric excipients [23]. The preparation of SRSDs can cause RES to be continuously released into the blood, reduce the influence of the quick metabolism and elimination of RES in the body, sustain the blood concentration of a treatment, and improve the bioavailability of RES. Furthermore, one-step preparation of a SRSD of poorly soluble and high-melting-point drugs through HME has rarely been reported in the literature until now. In this study, two polymers were used as carrier materials to control the release and prevent the photodegradation of RES. An SRSD of RES was prepared through one-step preparation of HME. The feasibility of HME in preparing high-melting-point and insoluble drug SRSDs was investigated.

## 2. Materials and Methods

### 2.1. Materials and Instruments

#### 2.1.1. Materials

Trans-RES with 99% purity (analytical grade) was obtained from Shanghai Aladdin Biochemical Technology Co., Ltd. (Shanghai, China). Eudragit RS (RS) was provided by Evonik Industries AG (Darmstadt, Germany). Kollidon 188 (P188) was donated by BASF Chemical Company (London, UK). Polyethylene glycol 6000 (PEG 6000) and PVP VA64 were purchased from Sinopharm Chemical Reagent Co., Ltd. (Shanghai, China). Other chemicals used were either of analytical or chromatographic grade.

Male Sprague-Dawley rats weighing approximately 220 g were purchased from Shanghai Slack Laboratory Animal Co., Ltd. (Shanghai, China). The animal certificate number is 20170005042863, the animal license is SCXK (Shanghai, China) 2017-0005, and the experiment was approved by Nanjing University of Chinese Medicine Animal Ethics Committee.

#### 2.1.2. Instruments

The following equipment were used: Pyris1 thermogravimetric analyzer (PerKin Elmer, Waltham, MA, USA), 200 F3 differential scanning calorimeter (NETZSCH group, Erlangen, Bayern, Germany), Wters2695 high-performance liquid chromatograph (Waters Corporation, Milford, MA, USA), CL21R Micro Bench Centrifuge (Thermo Fisher Scientific, Waltham, MA, USA), ZRS-8GD intelligent dissolution tester (Tiandatianfa, Tianjin, China), Pharma 11, co-rotating twin-screw extruder (Thermo Fisher Scientific), Bruker D8X X-ray diffractometer (Bruker Corporation, Billerica, MA, United States), Nicolet-iS10 Fourier transform infrared spectrometer (Thermo Fisher Scientific), Vortex Kylin-bell5, Vortex oscillator (Beideng, Nanjing, China), HY-45 air bath thermostatic shaker (JCGSYQ, Changzhou, China), JCM-7000 Scanning Electron Microscope (JEOL, Tokyo, Japan) and Huangcheng electric grinder (Wuyihaina, Jinhua, China).

### 2.2. Thermal Analysis

#### 2.2.1. Thermal Gravimetric Analysis (TGA)

The thermal stability of the samples was evaluated by TGA using Pyris l thermogravimetric analyzer. Samples (5–10 mg) were heated in aluminum pans to 500 °C at a heating rate of 10 °C·min^−1^. Nitrogen was used as purge gas at a flow rate of 30 mL·min^−1^.

#### 2.2.2. Differential Scanning Calorimetry (DSC)

Thermal behavior of the samples was examined by DSC. Each sample weighing approximately 6 mg was placed in open aluminum pans and heated over a temperature range of 20–300 °C at a linear heating rate of 10 °C·min^−1^, whereas nitrogen at a flow rate of 30 mL·min^−1^ was used as purge gas. The melting point was calculated as the midpoint of the peak. The instrument was calibrated using indium, and the data were analyzed with NETZSCH Proteus analysis software version 6.1 (NETZSCH group, Erlangen, Bayern, Germany).

### 2.3. Miscibility Study

The compatibility between drugs and carriers is usually evaluated using the solubility parameter method [24], which was originally employed in solvent-polymer systems proposed by Hildebrand in 1916. In 1999, Greenhalgh applied the Hildebrand solubility parameter method to prepare ibuprofen-carrier SDs [25]. Based on the Hildebrand solubility parameter method, the solubility parameter was divided into three parts by Hansen [26]. The formula is as follows:

(1) $$\delta^{2}=\delta_{d}^{2}+\delta_{P}^{2}+\delta_{h}^{2}$$

(2) $$\delta_{d}=\frac{{\Sigma E}_{\mathrm{di}}}{v}\;;\;\delta_{p}=\frac{\sqrt{\sum E_{\mathrm{Pi}}^{2}}}{V}$$

where, δ is the total solubility parameter, $\delta_{d}$ is the dispersion force solubility parameter, $\delta_{P}$ is the polar force solubility parameter, $\delta_{h}$ is the hydrogen bond solubility parameter, $E_{\mathrm{di}}$ is the group contribution of the dispersion force, $E_{\mathrm{Pi}}$ is the polar force, $E_{\mathrm{hi}}$ is the hydrogen bond, and V is the molar volume.

### 2.4. Drug Content

Methanol was used to dissolve the RES and SRSDs. Concentration of the samples was determined through a HPLC system with UV detection at 306 nm and a Hedra ODS-2 C18 column (250 nm × 4.6 nm, 5 μm). All measurements were performed with the injection volume of 10 μL. The mobile phase system consisted of the following: A (0.1% phosphoric acid in Milli Q water) and B (acetonitrile) with a flow rate of 1.0 mL·min^−1^ at a temperature of 35 °C and an organic phase-to-water ratio of 65:35.

Determination of RES in plasma samples: The chromatographic column was a Hedra OSD-2 C18 column (250 nm × 4.6 nm, 5 μm), the mobile phase methanol was the organic phase, the 0.1% phosphoric acid aqueous solution was the water phase, the ratio of organic phase to water was 65:35, and the temperature of the column oven was 35 °C. The measurement was performed at a flow rate of 1 mL·min^−1^, and the injection volume was 20 μL.

### 2.5. Preparation of SRSD

Accurately weighed quantities of RES, RS, and PEG6000 were mixed by a vortex for 3–5 min. The mixture was then manually added into the hopper, kneaded by the screw driving system, and finally extruded. During manufacture, the barrel temperature was preset and the screw speed was adjusted from low to high. After discharging, the extrudates were cooled by nitrogen and milled by a laboratory grinder. The obtained solid powder was passed through an 80-mesh sieve and stored in a desiccator (RH 65% ± 5%) at room temperature for further analysis.

Physical mixtures having the same composition of the SRSD were accurately weighed and then manually blended for approximately 5 min. All powders were manually screened through 80-mesh sieves.

#### Univariate Analysis

The content, crystallinity, and in vitro dissolution of RES were used as evaluation indicators. The effects of the above factors on the content and release of the SD were explored through univariate analysis on the mass ratio, the type and the amount of release modifier, the barrel temperature, and the screw speed of HME.

##### The Screening of Drug-Carrier Mass Ratio

RES and RS with mass ratios of 1:2, 1:3, 1:4, and 1:5 and PEG6000 with a mass fraction of 5% were accurately weighed and then vortex mixed for 3–5 min. The mixture was then manually added into the hopper, kneaded by the screw driving system, and finally extruded. The barrel temperature of the hot melt extruder was set at 170 °C, and the screw speed was set at 80 rpm for the experiment. The extrudates were cooled by nitrogen and milled by a laboratory grinder. The obtained solid powder was passed through an 80-mesh sieve and stored in a desiccator (RH 65% ± 5%) at room temperature for further analysis.

The content, crystallinity, and in vitro dissolution of RES were used as evaluation indicators to screen the best mass ratio.

##### The Screening of Release Modifier

RES and RS with the best mass ratio and (PEG6000, PVP VA, P188) with a mass fraction of 5% were accurately weighed. All samples were vortex mixed for 3–5 min. The mixture was then manually added into the hopper, kneaded by the screw driving system, and finally extruded. The barrel temperature of the hot melt extruder was set at 170 °C, and the screw speed was set at 80 rpm for the experiment. The extrudates were cooled by nitrogen and milled by a laboratory grinder. The obtained solid powder was passed through an 80-mesh sieve and stored in a desiccator (RH 65% ± 5%) at room temperature for further analysis.

The content, crystallinity, and in vitro dissolution of RES were used as evaluation indicators to screen the best release modifier.

##### The Screening of Amount of Release Modifier

RES and RS with the best mass ratio and the best release modifier with mass fractions of (5, 10, 15, 20, 25, 30, 35, 40, and 45%) were accurately weighed. All samples were vortex mixed for 3–5 min. The mixture was then manually added into the hopper, kneaded by the screw driving system, and finally extruded. The barrel temperature of the hot melt extruder was set at 170 °C, and the screw speed was set at 80 rpm for the experiment. The extrudates were cooled by nitrogen and milled by a laboratory grinder. The obtained solid powder was passed through an 80-mesh sieve and stored in a desiccator (RH 65% ± 5%) at room temperature for further analysis.

The content, crystallinity, and in vitro dissolution of RES were used as evaluation indicators to screen the best amount of release modifier.

##### The Screening of Barrel Temperature

RES and RS with the best mass ratio and the best release modifier with mass fraction of the best amount were accurately weighed. All samples were vortex mixed for 3–5 min. The mixture was then manually added into the hopper, kneaded by the screw driving system, and finally extruded. The barrel temperature of the hot melt extruder was set at 150 °C, 160 °C, 170 °C, 180 °C, and 190 °C, and the screw speed was set at 80 rpm for the experiment. The extrudates were cooled by nitrogen and milled by a laboratory grinder. The obtained solid powder was passed through an 80-mesh sieve and stored in a desiccator (RH 65% ± 5%) at room temperature for further analysis.

The content, crystallinity, and in vitro dissolution of RES were used as evaluation indicators to screen the best barrel temperature.

##### The Screening of Screw Speed

RES and RS with the best mass ratio and the best release modifier with mass fraction of the best amount were accurately weighed. All samples were vortex mixed for 3–5 min. The mixture was then manually added into the hopper, kneaded by the screw driving system, and finally extruded. The barrel temperature of the hot melt extruder was set at the best barrel temperature, and the screw speed was set at 40, 60, 80, 100, and 120 rpm for the experiment. The extrudates were cooled by nitrogen and milled by a laboratory grinder. The obtained solid powder was passed through an 80-mesh sieve and stored in a desiccator (RH 65% ± 5%) at room temperature for further analysis.

The content, crystallinity, and in vitro dissolution of RES were used as evaluation indicators to screen the best screw speed.

### 2.6. Saturated Solubility

The solubility of RES in pH 6.8 phosphate buffer was measured using a shake-flask method. Excess amount of RES was added to the above solutions, which were transferred to an air bath thermostatic shaker at 37 °C for 24 h. The solution was filtered through a 0.45 μm membrane filter and analyzed by HPLC.

### 2.7. In-Vitro Release Study

A dissolution apparatus was used to study the drug release of SDs. The studies were carried out at 37 °C ± 0.5 °C with a stirring speed of 75 rpm in 900 mL of dissolution medium (pH 6.8). Each formulation contained drug amount equivalent to 10 mg of RES. Samples were withdrawn at predetermined time intervals and replaced with medium of the same volume. The collected samples were filtered through 0.45 μm membrane filters and determined by HPLC. All dissolution tests were performed in triplicate. Three batches of SDs were prepared via the optimized preparation process, and the drug release profiles were similar.

### 2.8. FTIR

The FTIR spectra of the drug, selected excipients, physical mixture, and SD formulations were obtained using FTIR within the range of 400–4000 cm^−1^. The samples were powdered under nitrogen and mixed using the potassium bromide disk technique.

### 2.9. XRD

Powder X-ray diffraction was performed at room temperature with an X-ray diffractometer using Ni-filtered Cu K α radiation (voltage 40 kV, current 20 mA). The scanning rate was 2°·min^−1^ over a range of 5–50° and with an interval of 0.02°.

### 2.10. SEM

Morphology of the sample surface was characterized by scanning electron microscopy (SEM). The SDs were mounted on aluminum stubs using double-sided adhesive tape and then gold coated and examined using SEM.

### 2.11. Drug Release Kinetics

The release kinetics profiles were studied by fitting into zero-order, first-order, Higuchi, Ritger–Peppas, Weibull, and Hixson–Crowell models, as shown in Table 1 [27]. Each parameter was determined by linear least-squares fitting methods, and best fit was assessed by the correlation factor as R^2^.

### 2.12. Stability Study

Stability was affected by several factors, such as heat, light, and air [28]. In this article, humidity, temperature, and light were investigated systematically.

#### Influencing Factor Tests

##### High Humidity Test

An SRSD with a thickness of no more than 5 mm was placed in a flat weighing bottle and then placed in a constant-temperature and -humidity airtight dryer with the saturated KNO_3_ solution (RH 90% ± 5%) at the bottom. Within 10 days, the properties of the samples were observed, and the content of RES was determined.

##### Strong Light Exposure Test

An SRSD with a thickness of no more than 5 mm was placed in a flat weighing bottle and then placed under 4500 Lx ± 500 Lx light conditions. Within 10 days, the properties of the samples were observed, and the content of RES was determined.

##### High Temperature Test

An SRSD with a thickness of no more than 5 mm was placed in a flat weighing bottle and then placed in a constant-temperature (60 °C, 40 °C) and -humidity airtight dryer. Within 10 days, the properties of the samples were observed, and the content of RES was determined.

##### Long-Term Retention Test

On the basis of the results of influencing factor test, the samples were stored at room temperature (25 ± 2 °C, RH 65% ± 5%), tightly enclosed in a 50 mL plug tube for 6 months to assess the long-term stability of the prepared SD. The forms of RES were checked at 1, 2, 3, and 6 months.

### 2.13. In Vivo Study

Twelve male Sprague-Dawley rats were randomly divided into two groups (RES group and RES/RS/PEG6000 SRSD group) and orally administered by 0.5% CMC-Na suspension liquid of RES and SRSD (equivalent to 50 mg·kg^−1^ RES), respectively. Blood samples were collected at different times of 0.25, 0.5, 0.75, 1, 1.5, 2, 3, 4, 6, 8, 10, 12, and 24 h in EDTA tubes and then centrifuged at 3500 rpm for 10 min to separate the plasma. The plasma was placed in a 1.5 mL EP tube, naringenin (Nar) solution was added as an internal standard, and methanol was added to precipitate the protein content of plasma [29]. The samples were centrifuged at 13,000 rpm for 10 min in a centrifuge tube, and the supernatant was collected and stored at −20 °C until analysis. The concentration of plasma samples was determined in accordance with the in vivo drug concentration determination method. The DAS 3.0 pharmacokinetic program was applied to analyze the pharmacokinetic parameters.

## 3. Results and Discussion

### 3.1. Thermal Analysis

**Figure 1:**
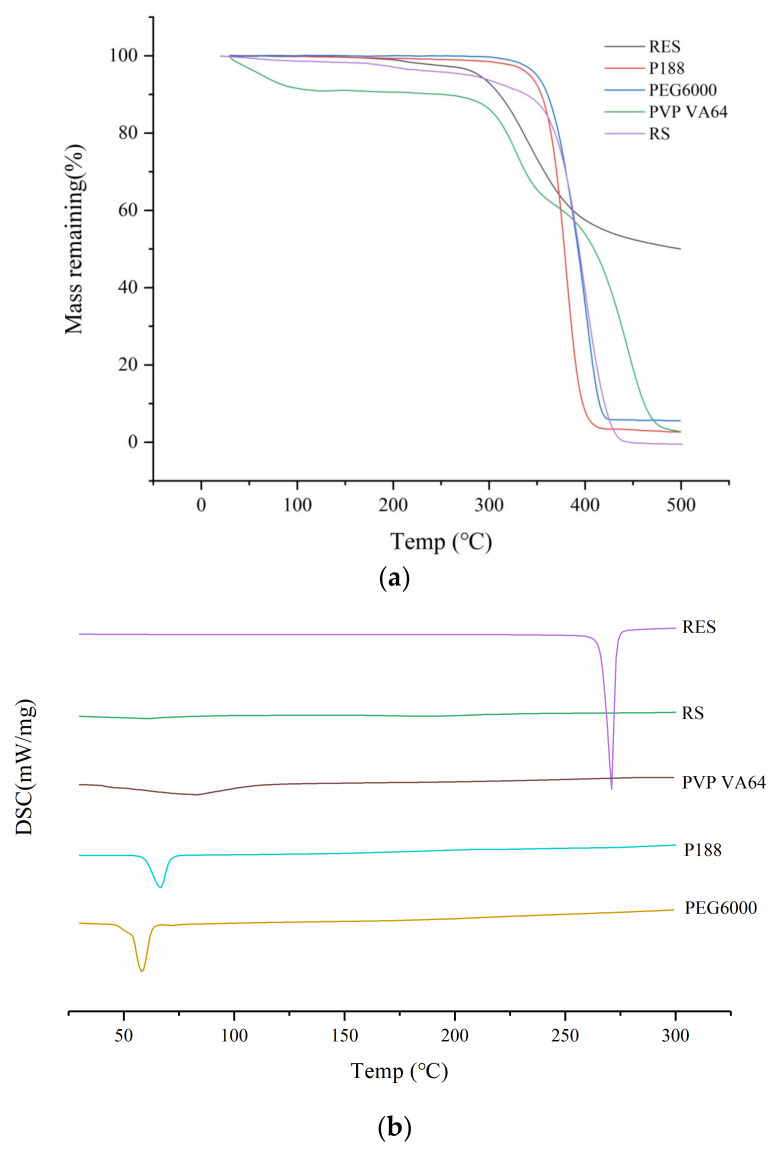
TGA diagram (**a**) and DSC diagram (**b**) of resveratrol and carriers.

The thermodynamic properties of the substance are important considerations in the preparation of SD via HME, which is closely related to melting and degradation. As shown in Figure 1a, RES and the excipients all exhibited good thermal stability, and thermal decomposition occurred after approximately 290 °C. In addition, the release modifier PVP VA64 had an obvious weight loss at 100 °C, which indicated that the excipient has a degree of hygroscopicity.

As illustrated in Figure 1b, a significant endothermic peak appeared near 266 °C, which corresponded to the melting point of RES. Eudragit RS is an amorphous form, playing a role of slow-release, which has a glass transition temperature of 62 °C. A broad peak appeared at approximately 200 °C, which is the characteristic melting peak of the polymeric material. The glass transition temperature of PVP VA64 was 103 °C. The melting points of PEG6000 and P188 were 70 °C and 62 °C, respectively.

### 3.2. Miscibility Study

Related literature studies have shown that a solubility parameter difference Δδ < 7 MPa^1/2^ indicates that the drug and the carrier have good compatibility in the molten state. By contrast, a solubility parameter difference Δδ > 10 MPa^1/2^ may indicate incompatibility between the drug and the carrier [30]. The solubility parameter values obtained using the group contribution method of RES and each carrier are shown in the Table 2. The solubility parameter values between RES and RS, PVP VA64, P188, and PEG6000 were all less than 7 MPa^1/2^, indicating that they all have a good miscibility [31].

### 3.3. Preparation of SRSD

#### Univariate Analysis

Univariate analysis of the drug–carrier mass ratio, the type and the amount of release modifier, the barrel temperature, and the screw speed of HME was conducted. The results are shown in Table 3.

##### The Effect of Drug-Carrier Mass Ratio

**Figure 2:**
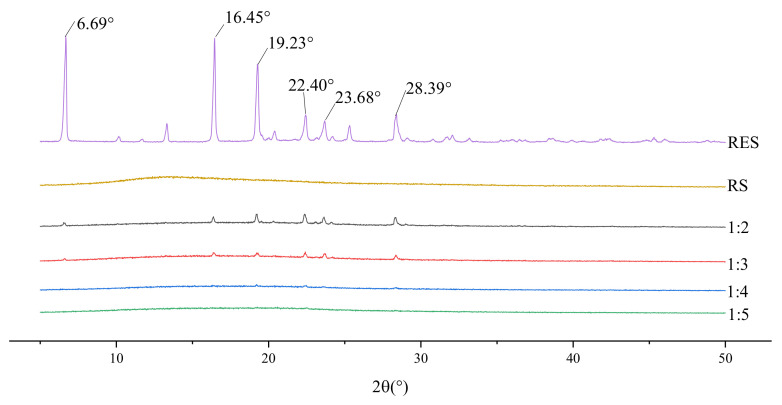
XRD diagram of solid dispersion prepared by different drug loading ratios.

**Figure 3:**
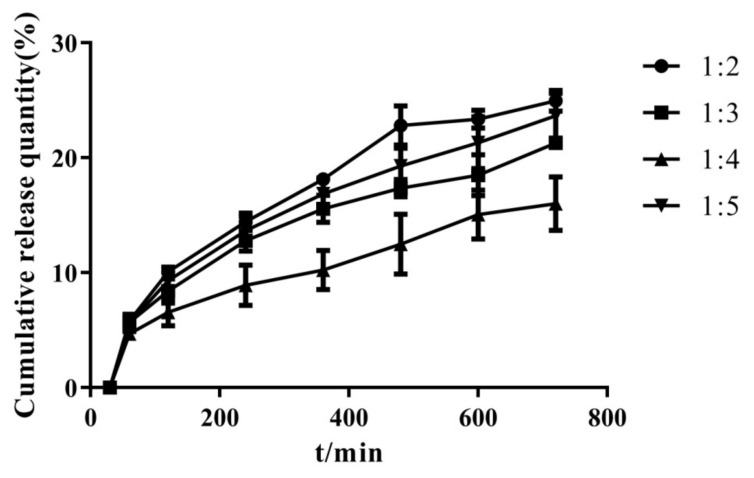
Dissolution curves of solid dispersion prepared by different drug loading ratios.

Figure 2 shows that the drug still existed in the form of partial microcrystals when the drug–carrier mass ratio was 1:2 or 1:3. When the proportion of the carrier gradually increased, the drug was highly dispersed in the carrier almost in an amorphous or molecular form. On the basis of the dissolution curves shown in Figure 3, the cumulative release of the drug was higher than those of the others when the drug–carrier ratio was 1:2. The drug existed in the carrier in microcrystalline form, indicating poor stability. Therefore, the follow-up test was selected under the condition that the dissolution was higher and the drug–carrier mass ratio was 1:5.

##### The Effect of Release Modifier

The use of the release modifier (water-soluble carrier) can effectively improve the release of the drug in the SRSD [32], and the selection of different types of release modifier greatly influenced drug release. After determining the drug-carrier ratio, the effects of different release modifiers on drug release were compared using poloxamer (P188), povidone (PVP), and polyethylene glycol (PEG6000).

**Figure 4:**
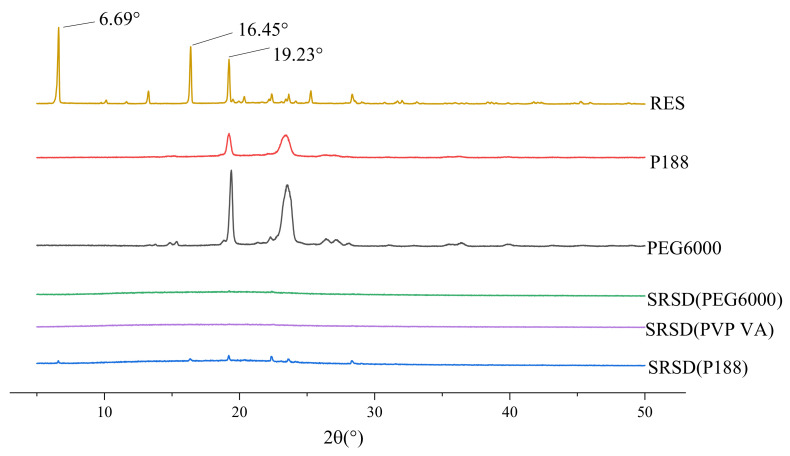
XRD diagram of solid dispersion prepared by different release modifiers.

**Figure 5:**
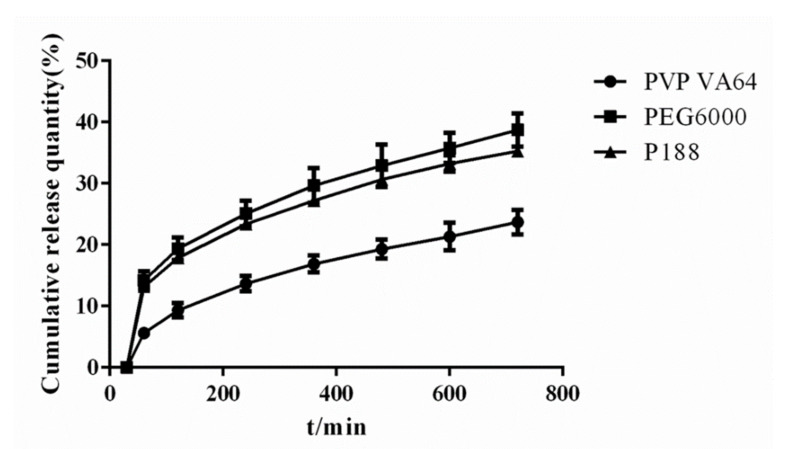
Dissolution curves of solid dispersion prepared by different release modifiers.

As shown in Figure 4, when the drug-carrier mass ratio was 1:5, the drug crystal diffraction peaks almost disappeared in the SRSD prepared with different release modifiers. As shown in Figure 5, When the drug-carrier mass ratio was 1:5, the dissolution profiles of P188 and PEG6000 were relatively similar. When the drug-carrier mass ratio was 1:5 and the release modifier was PEG6000, the cumulative drug release rate was the highest. Thus, PEG6000 was selected as the release modifier for subsequent experiments.

##### The Effect of Amount of Release Modifier

**Figure 6:**
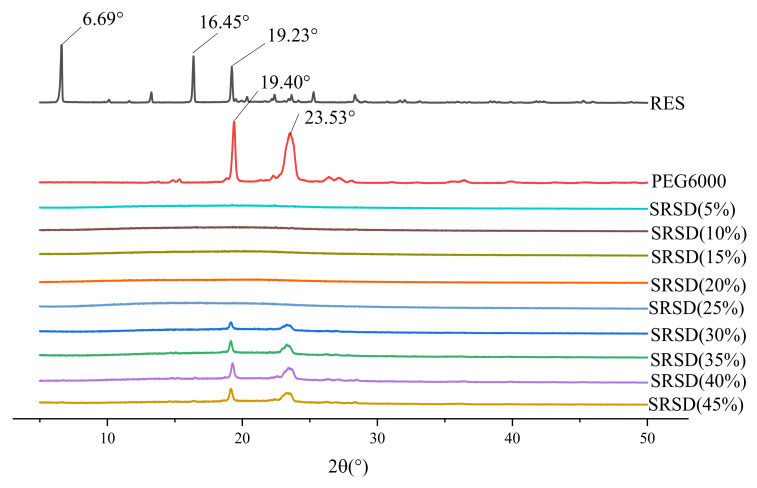
XRD diagram of solid dispersion prepared by different amount of release modifier.

**Figure 7:**
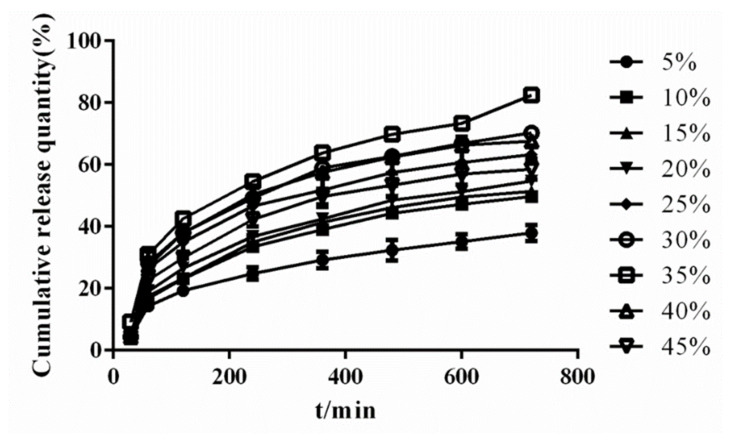
Dissolution curves of solid dispersion prepared by different amount of release modifier.

The XRD diagram is shown in Figure 6. The drug was in amorphous form at each mass ratio of release modifier. When the ratio of PEG6000 gradually increased to 30%, the characteristic crystal peak of PEG6000 appeared, but no drug crystal diffraction peak was found in the system, indicating that the crystalline drug was transformed into amorphous and had a high degree dispersion in carrier. The dissolution result is shown in Figure 7. The release rate of the drug enhanced with increasing release modifier. When the water–soluble carrier ratio was 35%, the drug release reached the highest level. When the ratio of release modifier continued to increase, the drug release may be restricted, which may be caused by the high viscosity of increase in water-soluble carrier [33]. Therefore, 35% was chosen as the ratio of release modifier.

##### The Effect of Barrel Temperature and Screw Speed

As a continuous process, processing parameters of HME play an important role in controlling the quality of the extrudates [34]. Several reports have illustrated the significant impact of processing parameters on the physicochemical properties of the SDs [35,36]. Among them, barrel temperature and screw speed are primary and vital process parameters. In the study, the cumulative drug release rate was used as an indicator to investigate the effect of operation parameters on the quality of the SRSDs.

**Figure 8:**
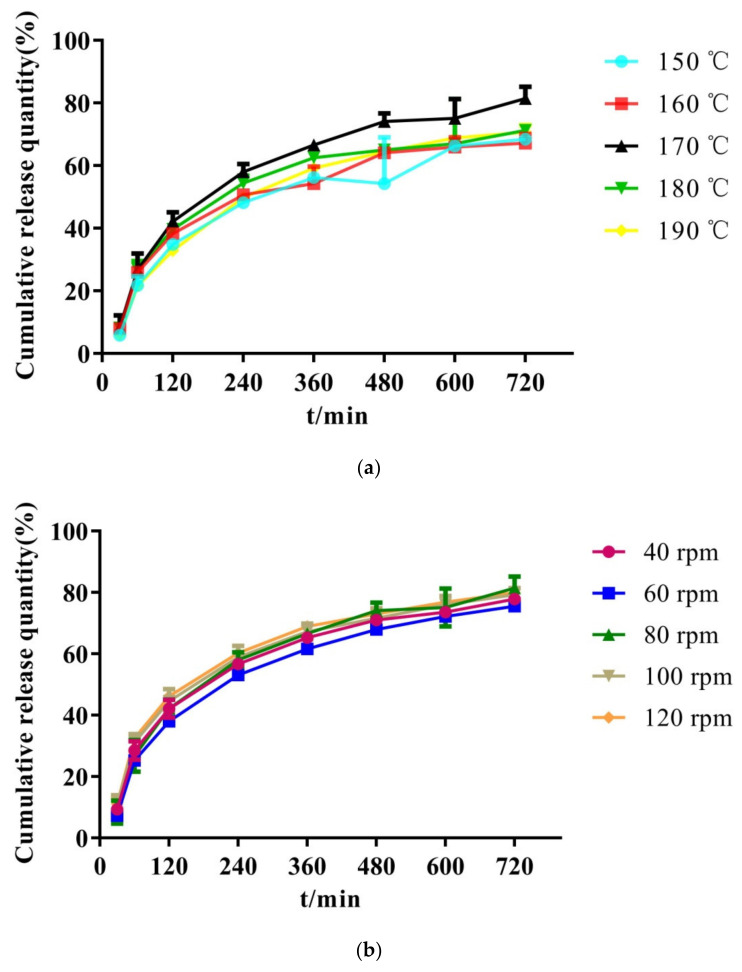
Dissolution curves of solid dispersion prepared by different barrel temperature (**a**) and screw speed (**b**).

As shown in Figure 8a, the energy provided by the barrel increased and the crystalline drug was more easily melted and dispersed in the carriers as the barrel temperature was increased. When the barrel temperature was 170 °C, the cumulative dissolution rate of the drug was the highest. When the temperature continued to rise, the cumulative dissolution rate of the drug in the SRSD decreased, which may be due to thermal degradation of overheating [37]. Thus, the preparation temperature of 170 °C was selected for the next experiment step.

The dissolution effect of different screw speeds on the preparation of SDs is not obvious in Figure 8b. When using the HME technology to prepare SDs, a slow screw speed may lead to excessive accumulation of materials and uneven mixing of the materials [38]. When the screw speed was excessively high, the residence time of the drug in the barrel was too short, and the drug may be insufficiently heated. Thus, 80 rpm was selected as the operating speed.

### 3.4. Saturated Solubility

As shown in Table 4, the saturated solubility was 2.28-fold higher than the bulk RES. RES was prepared as an SD and highly dispersed in the carriers. High dispersion and large specific surface area made it easier to dissolve and release in vitro.

### 3.5. In-Vitro Release Study

**Figure 9:**
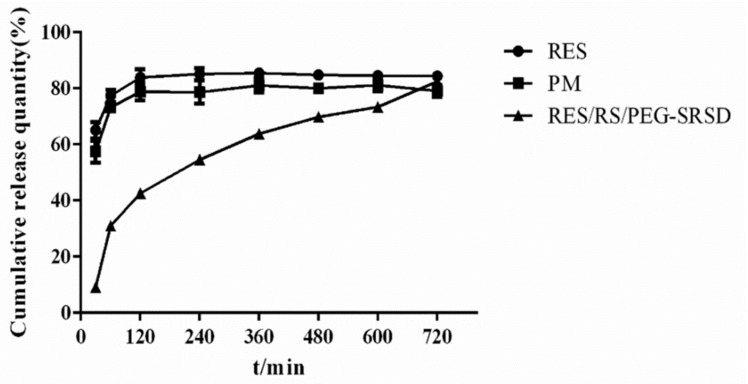
Dissolution curves of RES, physical mixture, and SRSD.

**Figure 10:**
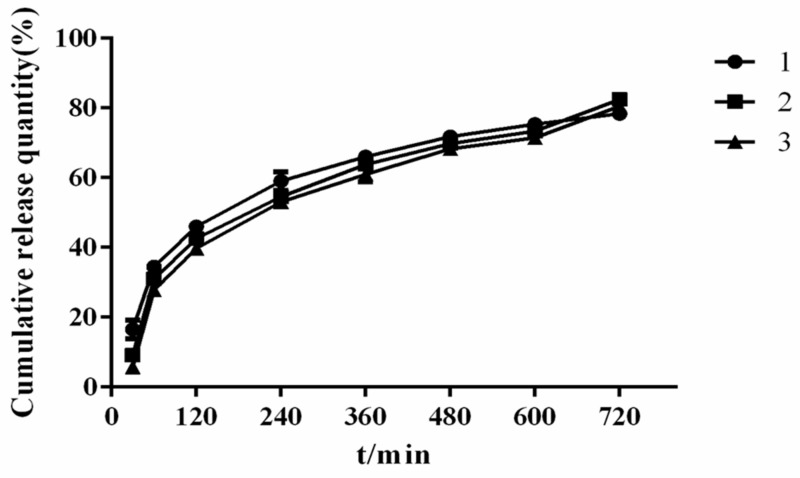
Dissolution curves of three batches of SRSDs.

As shown in Figure 9, compared with the raw materials and PM, the prepared RES/RS/PEG6000-SRSD exerted an obvious SR effect. Embedding a drug within an insoluble matrix is a convenient way of controlling the drug release [39]. The cumulative in vitro release reached approximately 82.42% at 12 h. Verification experiment results in Figure 10 showed that the three batches of SDs prepared exerted good SR effects, and the preparation process was stable and feasible.

### 3.6. FTIR

**Figure 11:**
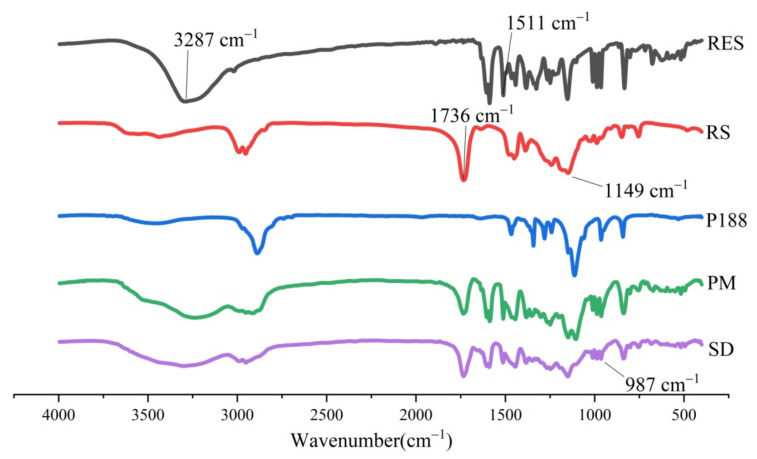
IR spectrum of RES, carriers, PM, and RES/RS/PEG6000-SRSD.

The spectra of RES, Eudragit RS (RS), PEG6000 (PEG6000), physical mixture (PM), and SD are illustrated in Figure 11. RES has a phenolic hydroxyl stretching vibration peak at 3287 cm^−1^ and a benzene ring absorption peak at 1511 cm^−1^. Two sharp peaks at 1736 and 1149 cm^−1^ were assigned to the carbonyl groups and C-O-C stretching vibration of RS, respectively [40]. In the spectrum of PM, the characteristic absorption peaks of the drug and excipients were retained, implying that no significant interaction occurred between the drug and excipients. In addition, a trans-carbon absorption peak (987 cm^−1^) appeared in the RES/RS/PEG6000-SRSD spectrum, indicating that RES still maintains a trans-space configuration and has biological activity in the prepared SD.

### 3.7. XRD

**Figure 12:**
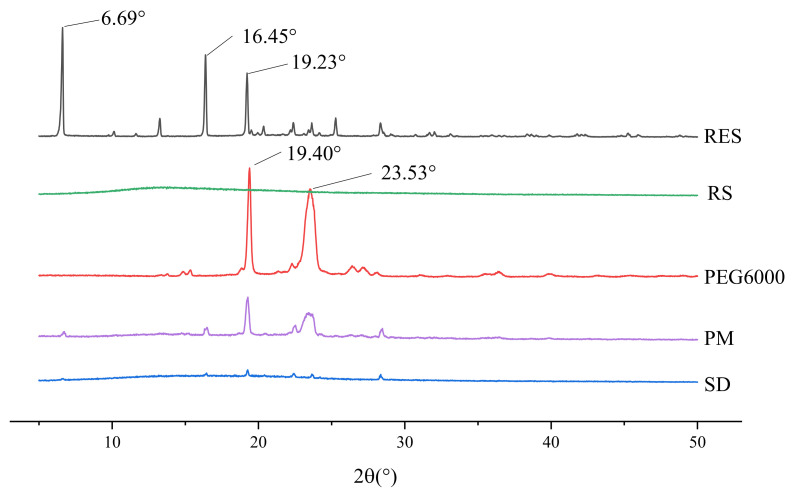
XRD spectrum of RES, carriers, PM, and RES/RS/PEG6000-SRSD.

As shown in Figure 12, the diffraction pattern of RES between 5° and 30° showed multiple narrow peaks, indicating highly crystalline structures. The polymer carrier RS was shown as an amorphous polymer. PEG6000 exhibited two characteristic diffraction peaks at 19.40° and 23.53°, respectively, indicating that it is a crystalline low-molecular substance. The characteristic superimposed peaks of RES and excipients appeared in the spectrum of PM, and the intensity decreased. Absence of the peaks distinct to the raw RES diffraction pattern of RES/RS/PEG6000 SRSD suggested a phase transition occurrence from the crystalline form to the amorphous one in the precipitation process.

### 3.8. SEM

**Figure 13:**
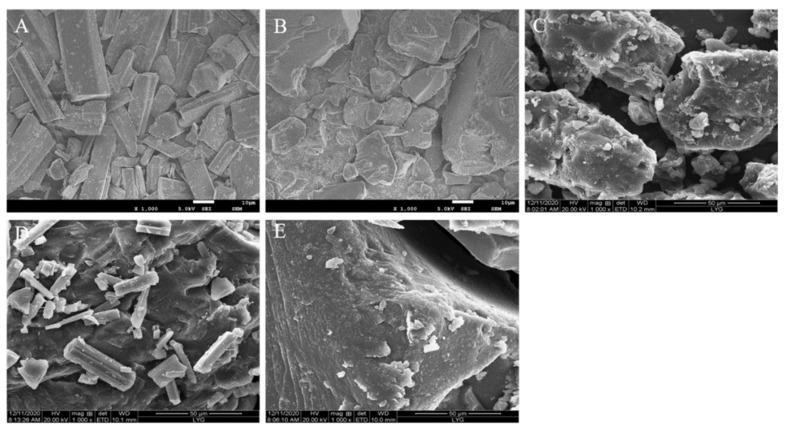
SEM microscopy of RES (**A**), RS (**B**), PEG6000 (**C**), PM (**D**), and RES/RS/PEG6000-SRSD (**E**).

The morphology of drug, carriers, and SD systems was checked through SEM (Figure 13). The bulk RES existed in the form of columnar crystals, the carrier RS existed in irregular lumps, and the PEG6000 existed in a smooth spherical shape. The PM contained RES in the form of columnar crystals and the carriers (RS and PEG6000) in irregular shapes. No crystals were found in the SD, suggesting that RES existed in the carrier in an amorphous or molecular state.

### 3.9. Drug Release Kinetics

On the basis of the result of the release pattern in Table 5, the correlation coefficient R^2^ of the Weibull Model was the highest of release kinetics profiles. The Weibull model is an empirical model, which is widely used in rapid-release and SR drug release systems [39]. Curve characteristic parameter β was obtained via the fitting. When β > 1, the sustained-release curve diagram appeared as an upward S-shape curve. When β < 1, it appeared as a curve with a steep initial slope. The cumulative release rate of RES in the phosphate buffer (pH 6.8) at 37 °C reached 80% within 2 h, and an obvious burst release phenomenon occurred. With the preparation of SRSD, the burst release of the RES improved and the RES/RS/PEG6000 SRSD showed a good sustained-release effect in 12 h.

From the sustained-release curve diagram, the polymer swelled and the drug dissolved in the medium and release diffused during the initial dissolution stage. When the swelling and dissolution slowed down, the drug was mainly diffused and released under the concentration gradient. At this time, the diffused release was the main process of drug release. DSC and XRD results showed that the drug existed in an amorphous or molecular state. It can increase the surface area of the drug, and a certain concentration of excipients effectively inhibited the formation of drug crystal nucleus.

In the Ritger–Peppas model, the diffusion index *n* value also reflected the drug release mechanism. For spherical pellets, *n* < 0.45 corresponds to Fick diffusion, 0.45 < *n* < 0.89 corresponds to the synergistic effect of diffusion and erosion, and *n* > 0.89 corresponds to erosion. Eudragit RS is a water-insoluble carrier. It can swell and contact with liquid in the gastrointestinal tract to form pores. The drug is released through the pores and release control is achieved. The diffusion index *n* value of the prepared RES/RS/PEG6000-SRSD was between 0.45 and 0.89, indicating that the release of the SD coexists with diffusion and erosion [41].

### 3.10. Stability Study

#### Influencing Factor Tests

##### High Humidity Test

As shown in Table 6 and Table 7, under high-humidity saturated KNO_3_ saturated solution (RH 90% ± 5%), the prepared RES/RS/PEG6000-SRSD had a high moisture absorption rate and a low content. Therefore, placing it in a saturated NaCl solution (RH 75% ± 5%) for 10 days was investigated. With the extension of storage time, the SD was slightly agglomerated and had hygroscopicity. In addition, the content decreased, which was higher than the Pharmacopoeia hygroscopicity requirement. Thus, RES-RS-PEG6000-SRSD needs to be sealed and stored under low-humidity conditions.

##### Strong Light Exposure Test

Table 8 shows that under strong light irradiation, RES/RS/PEG6000-SRSD exerted almost no effect on the short-term properties and content. The presence of the carrier improved the photosensitivity of RES.

##### High Temperature Test

As shown in Table 9 and Table 10, RES-RS-PEG6000-SRSD agglomerated under high temperature conditions (60 °C), which affected the weighing. Therefore, the high temperature test was further carried out at 40 °C. When the storage temperature was 40 °C, SRSD showed good stability.

##### Long-Term Retention Test

**Figure 14:**
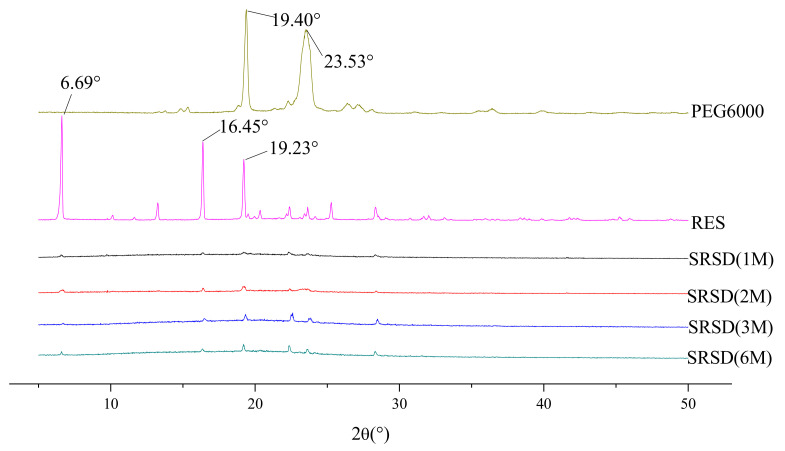
XRD diagram of RES/RS/PEG6000-SRSD at different times.

The long-term stability of the SRSDs was determined by XRD. As shown in Figure 14, no recrystallization of the amorphous drug occurred in the SRSDs, suggesting that it had good physical stability.

### 3.11. In Vivo Study

**Figure 15:**
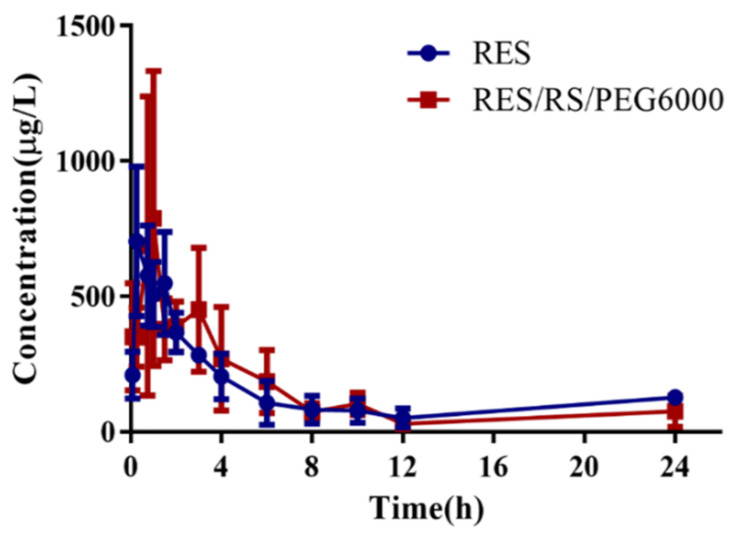
Plasma concentration-time profiles of RES and RES/RS/PEG6000 after drug administration in rats.

The in vivo results (Figure 15 and Table 11) showed that the T_max_ values of the RES and RES/RS/PEG6000 SRSD were 0.79 and 1.54 h, respectively. Given the preparation of SRSD, the t_1/2z_ of RES/RS/PEG6000 SRSD was longer than that of RES. The C_max_ of RES/RS/PEG6000 SRSD was 815.65 µg·L^−1^, which was higher than that of RES, which may be caused by the sustained absorption. In addition, the AUC_0–∞_ values were 2879.75 and 4042.63 μg·L^−1^·h^−1^, respectively. The bioavailability of RES/RS/PEG6000 SRSD relative to the raw RES was 140.38%, combined with the increase in saturation solubility, which indicated that the SD technology can improve solubility and bioavailability [40,42,43].

## 4. Conclusions

RES SRSD was successfully prepared by HME. Amorphous RES was highly dispersed into the carriers, and the solubility of RES was increased 2.28 times. The sustained-release profiles of RES followed a Weibull mode kinetic. Compared with RES, RES/RS/PEG6000 SRSD had a significantly prolonged half-life from 3.8 to 7.1 h and a 1.4-fold increased bioavailability. Univariate analysis suggested that the concentration of release modifier played an important role in controlling the release of RES. The use of RS is responsible for substantially reducing the release rate of drugs. The high-melting-point and insoluble drug SRSDs were prepared by adjusting the formulation and processing of HME. The above results conformed such systems into a versatile tool that could potentially endow therapeutic benefits in the treatment through prolonged retention and delivery. Further study will be continued to increase the solubility and bioavailability of RES and to analyze drug release at different temperatures.

## Figures and Tables

**Table 1:** Release model equations.

Model	Equation
Zero order Model	M_t_/M_∞_ = k*t*
First order Model	ln (1 − M_t_/M_∞_) = −k*t*
Higuchi Model	M_t_/M_∞_ = k*t*^1/2^
Ritger-Peppas Model	M_t_/M_∞_ = k*t^n^*
Weibull Model	M_t_/M_∞_ = 1 − e(−α*t*^β^)
Hixson-Crowell Model	(1 − M_t_/M_∞_)^1/3^ = k*t*

Where M*_t_*/M_∞_ is the cumulative drug release rate in *t* time, k is the release rate constant, and *n* is the diffusion index, which is the characteristic parameter characterizing the release mechanism, α is a scale parameter, β is the characteristic parameter of curve character.

**Table 2:** The solubility parameter values for drugs and carriers.

Sample	δ_d_ (MPa^1/2^)	δ_p_ (MPa^1/2^)	δ_h_ (MPa^1/2^)	δ (MPa^1/2^)	Δδ (MPa^1/2^)
RES	20.9	5.2	5.9	22.3	-
Eudragit RS	16.94	1.02	11.26	20.4	1.9
PVP VA64	17.4	0.5	9.2	19.7	2.6
P188	16.4	6.9	5.8	18.7	3.6
PEG6000	17.78	11.11	9.13	22.9	0.6

**Table 3:** Univariate analysis of SRSD.

No.	Mass Ratio	Release Modifier	Amount of Release Modifier (%)	Barrel Temperature (°C)	Screw Speed (rpm)	Content (%)
1	1:2	PEG6000	5	170	80	95.55
2	1:3	PEG6000	5	170	80	96.27
3	1:4	PEG6000	5	170	80	97.32
4	1:5	PEG6000	5	170	80	95.28
5	1:5	P188	5	170	80	96.39
6	1:5	PVP VA64	5	170	80	98.50
7	1:5	PEG6000	10	170	80	98.87
8	1:5	PEG6000	15	170	80	95.89
9	1:5	PEG6000	20	170	80	95.30
10	1:5	PEG6000	25	170	80	95.72
11	1:5	PEG6000	30	170	80	101.50
12	1:5	PEG6000	35	170	80	101.28
13	1:5	PEG6000	40	170	80	95.03
14	1:5	PEG6000	45	170	80	96.38
15	1:5	PEG6000	35	150	80	95.66
16	1:5	PEG6000	35	160	80	95.12
17	1:5	PEG6000	35	180	80	97.04
18	1:5	PEG6000	35	190	80	98.00
19	1:5	PEG6000	35	170	40	97.56
20	1:5	PEG6000	35	170	60	96.96
21	1:5	PEG6000	35	170	100	101.22
22	1:5	PEG6000	35	170	120	102.38

**Table 4:** Saturated solubility of RES and RES/RSPEG6000-SRSD.

	RES	RES/RS/PEG6000-SRSD	Fold
Saturated solubility (μg·mL^−1^)	44.53 ± 0.29	101.65 ± 16.98	2.28

**Table 5:** Release pattern of RES-SD.

Model	Equation	R^2^
Zero order model	Q*_t_* = 5.43 × *t* + 21.37	0.8389
First order model	Q*_t_* = 76.23 × (1 − e − 0.32*t*)	0.9590
Higuchi model	Q*_t_* = 24.00 × *t*^1/2^ − 0.1294	0.9388
Ritger–Peppas model	Q*_t_* = 25.32 × *t*^0.4720^	0.9411
Weibull model	Q*_t_* = 84.085 × [1 − e(−3.129 × *t*^0.82^)]	0.9592
Hixson–Crowell model	Q*_t_* = 100 × [1 − (1 − 0.043 × *t*)^3^]	0.8249

Q*_t_* is the released drug amount at time (*t*).

**Table 6:** The characteristic and moisture absorption under high humidity (saturated KNO_3_ solution, RH 90% ± 5%).

T/d	Characteristic	Content	Moisture Absorption Rate	RSD of Content	RSD of Moisture Absorption Rate
0	White and off-white powder	99.83%	0%	0.08%	0
5	Powder aggregation	93.91%	17.46%	0.22%	0.58%
10	Powder aggregation	86.82%	7.28%	1.00%	1.21%

**Table 7:** The characteristic and moisture absorption under high humidity (saturated NaCl solution, RH 75% ± 5%).

T/d	Characteristic	Content	Moisture Absorption Rate	RSD of Content	RSD of Moisture Absorption Rate
0	White and off-white powder	95.95%	0%	0.07%	0
5	Powder aggregation slightly	97.29%	5.93%	0.26%	0.63%
10	Powder aggregation slightly	99.47%	3.27%	0.98%	1.26%

**Table 8:** The character and content under strong light.

	T/d	Characteristic	Content
RES	0	White and off-white powder	98.39%
	5	Light yellow powder	95.78%
	10	Light yellow powder	86.42%
SRSD	0	White and off-white powder	99.83%
	5	White and off-white powder	99.81%
	10	White and off-white powder	98.02%

**Table 9:** The character and content under high temperature conditions (60 °C).

T/d	Characteristic	Content
0	White and off-white powder	99.83%
5	Brown agglomerates	/
10	Brown agglomerates	/

**Table 10:** The character and content under high temperature conditions (40 °C).

T/d	Characteristic	Content
0	White and off-white powder	95.95%
5	White and off-white powder	95.70%
10	White and off-white powder	95.75%

**Table 11:** Pharmacokinetic parameters of raw RES and SRSDs in rats after oral administration (*n* = 6).

Parameters	Raw RES	RES/RS/PEG6000
AUC_0–t_ (μg·L^−1^·h^−1^)	2744.80 ± 709.21	3298.84 ± 1345.36
AUC_0–∞_ (μg·L^−1^·h^−1^)	2879.75 ± 546.56	4042.63 ± 2049.90
t_1/2z_(h)	3.78 ± 2.80	7.09 ± 6.20
T_max_ (h)	0.79 ± 0.64	1.54 ± 0.82
C_max_ (μg·L^−1^)	681.95 ± 217.31	815.65 ± 366.89

## Data Availability

The raw data is available from the authors upon request.

## Sample Availability

Samples of the compounds are not available from the authors.

## References

[1] Chen R., Moriya J., Yamakawa J., Takahashi T., Kanda T. (2010). Traditional chinese medicine for chronic fatigue syndrome. Evid. Based Complement. Alternat. Med..

[2] Liu X., Wang Y., Ji H., Aihara K., Chen L. (2016). Personalized characterization of diseases using sample-specific networks. Nucleic Acids Res..

[3] Kim D., Shih C.C., Cheng H.C., Kwon S.H., Kim H., Lim B. (2021). A comparative study of the traditional medicine systems of South Korea and Taiwan: Focus on administration, education and license. Integr. Med. Res..

[4] Dong X., Fu J., Yin X., Cao S., Li X., Lin L., Ni J. (2016). Emodin: A Review of its Pharmacology, Toxicity and Pharmacokinetics. Phytother. Res..

[5] Jardim F.R., De Rossi F.T., Nascimento M.X., Da Silva Barros R.G., Borges P.A., Prescilio I.C., De Oliveira M.R. (2018). Resveratrol and Brain Mitochondria: A Review. Mol. Neurobiol..

[6] Xia N., Daiber A., Förstermann U., Li H. (2017). Antioxidant effects of resveratrol in the cardiovascular system. Br. J. Pharmacol..

[7] Li W., Quan P., Zhang Y., Cheng J., Liu J., Cun D., Xiang R., Fang L. (2014). Influence of drug physicochemical properties on absorption of water insoluble drug nanosuspensions. Int. J. Pharm..

[8] Vasconcelos T., Marques S., Das Neves J., Sarmento B. (2016). Amorphous solid dispersions: Rational selection of a manufacturing process. Adv. Drug Deliv. Rev..

[9] Nivelle L., Hubert J., Courot E., Jeandet P., Aziz A., Nuzillard J.M., Renault J.H., Clément C., Martiny L., Delmas D. (2017). Anti-Cancer Activity of Resveratrol and Derivatives Produced by Grapevine Cell Suspensions in a 14 L Stirred Bioreactor. Molecules.

[10] Wang W., Zhang L., Chen T., Guo W., Bao X., Wang D., Ren B., Wang H., Li Y., Wang Y. (2017). Anticancer Effects of Resveratrol-Loaded Solid Lipid Nanoparticles on Human Breast Cancer Cells. Molecules.

[11] Huang Y., Dai W.G. (2014). Fundamental aspects of solid dispersion technology for poorly soluble drugs. Acta Pharm. Sin. B.

[12] Mori Y., Motoyama K., Ishida M., Onodera R., Higashi T., Arima H. (2019). Theoretical and practical evaluation of lowly hydrolyzed polyvinyl alcohol as a potential carrier for hot-melt extrusion. Int. J. Pharm..

[13] Chowdhury N., Vhora I., Patel K., Bagde A., Kutlehria S., Singh M. (2018). Development of Hot Melt Extruded Solid Dispersion of Tamoxifen Citrate and Resveratrol for Synergistic Effects on Breast Cancer Cells. AAPS PharmSciTech.

[14] Repka M.A., Bandari S., Kallakunta V.R., Vo A.Q., Mcfall H., Pimparade M.B., Bhagurkar A.M. (2018). Melt extrusion with poorly soluble drugs—An integrated review. Int. J. Pharm..

[15] Shah S., Maddineni S., Lu J., Repka M.A. (2013). Melt extrusion with poorly soluble drugs. Int. J. Pharm..

[16] Palazi E., Karavas E., Barmpalexis P., Kostoglou M., Nanaki S., Christodoulou E., Bikiaris D.N. (2018). Melt extrusion process for adjusting drug release of poorly water soluble drug felodipine using different polymer matrices. Eur. J. Pharm. Sci. Off. J. Eur. Fed. Pharm. Sci..

[17] Lakshman J.P., Cao Y., Kowalski J., Serajuddin A.T. (2008). Application of melt extrusion in the development of a physically and chemically stable high-energy amorphous solid dispersion of a poorly water-soluble drug. Mol. Pharm..

[18] Bennett R.C., Brough C., Miller D.A., O’Donnell K.P., Keen J.M., Hughey J.R., Williams R.O., Mcginity J.W. (2015). Preparation of amorphous solid dispersions by rotary evaporation and KinetiSol Dispersing: Approaches to enhance solubility of a poorly water-soluble gum extract. Drug Dev. Ind. Pharm..

[19] Hörmann T.R., Rehrl J., Scheibelhofer O., Schaden L.M., Funke A., Makert C., Khinast J.G. (2019). Sensitivity of a continuous hot-melt extrusion and strand pelletization line to control actions and composition variation. Int. J. Pharm..

[20] Evans R.C., Bochmann E.S., Kyeremateng S.O., Gryczke A., Wagner K.G. (2019). Holistic QbD approach for hot-melt extrusion process design space evaluation: Linking materials science, experimentation and process modeling. Eur. J. Pharm. Biopharm..

[21] Solanki N.G., Gumaste S.G., Shah A.V., Serajuddin A.T.M. (2019). Effects of Surfactants on Itraconazole-Hydroxypropyl Methylcellulose Acetate Succinate Solid Dispersion Prepared by Hot Melt Extrusion. II: Rheological Analysis and Extrudability Testing. J. Pharm. Sci..

[22] Huang B.B., Liu D.X., Liu K., Wu G. (2019). Application of Solid Dispersion Technique to Improve Solubility and Sustain Release of Emamectin Benzoate. Molecules.

[23] Lee Y.S., Song J.G., Lee S.H., Han H.K. (2017). Sustained-release solid dispersion of pelubiprofen using the blended mixture of aminoclay and pH independent polymers: Preparation and in vitro/in vivo characterization. Drug. Deliv..

[24] Mohammad M.A., Alhalaweh A., Velaga S.P. (2011). Hansen solubility parameter as a tool to predict cocrystal formation. Int. J. Pharm..

[25] Greenhalgh D.J., Williams A.C., Timmins P., York P. (1999). Solubility parameters as predictors of miscibility in solid dispersions. J. Pharm. Sci..

[26] Hansen C.M. (2004). 50 Years with solubility parameters—Past and future. Prog. Org. Coat..

[27] Tamayo A., Mazo M.A., Veiga M.D., Ruiz-Caro R., Notario-Pérez F., Rubio J. (2017). Drug kinetics release from Eudragit—Tenofovir@SiOC tablets. Mater. Sci. Eng. C Mater. Biol. Appl..

[28] Spanopoulos I., Hadar I., Ke W., Tu Q., Chen M., Tsai H., He Y., Shekhawat G., Dravid V.P., Wasielewski M.R. (2019). Uniaxial Expansion of the 2D Ruddlesden-Popper Perovskite Family for Improved Environmental Stability. J. Am. Chem. Soc..

[29] Kumar S., Lather V., Pandita D. (2016). Stability indicating simplified HPLC method for simultaneous analysis of resveratrol and quercetin in nanoparticles and human plasma. Food Chem..

[30] Tsakiridou G., Reppas C., Kuentz M., Kalantzi L. (2019). A Novel Rheological Method to Assess Drug-Polymer Interactions Regarding Miscibility and Crystallization of Drug in Amorphous Solid Dispersions for Oral Drug Delivery. Pharmaceutics.

[31] Ning Y., Cheng-Hui H., Wei H., Jian-Guo X., Xuan-Lin L. (2014). Application of hot-melt extrusion technique in pharmaceutical research. J. Int. Pharm. Res..

[32] Park J.B., Lee B.J., Kang C.Y., Repka M.A. (2017). Process Analytical Quality Control of Tailored Drug Release Formulation Prepared via Hot-Melt Extrusion Technology. J. Drug Deliv. Sci. Technol..

[33] Alshahrani S.M., Morott J.T., Alshetaili A.S., Tiwari R.V., Majumdar S., Repka M.A. (2015). Influence of degassing on hot-melt extrusion process. Eur. J. Pharm. Sci. Off. J. Eur. Fed. Pharm. Sci..

[34] Weuts I., Kempen D., Six K., Peeters J., Verreck G., Brewster M., Van Den Mooter G. (2003). Evaluation of different calorimetric methods to determine the glass transition temperature and molecular mobility below T(g) for amorphous drugs. Int. J. Pharm..

[35] Liu H., Wang P., Zhang X., Shen F., Gogos C.G. (2010). Effects of extrusion process parameters on the dissolution behavior of indomethacin in Eudragit E PO solid dispersions. Int. J. Pharm..

[36] Reitz E., Podhaisky H., Ely D., Thommes M. (2013). Residence time modeling of hot melt extrusion processes. Eur. J. Pharm. Biopharm. Off. J. Arb. Pharm. Verfahr..

[37] Kashif P.M., Madni A., Ashfaq M., Rehman M., Mahmood M.A., Khan M.I., Tahir N. (2017). Development of Eudragit RS 100 Microparticles Loaded with Ropinirole: Optimization and In Vitro Evaluation Studies. AAPS PharmSciTech.

[38] Ghaffari A., Navaee K., Oskoui M., Bayati K., Rafiee-Tehrani M. (2007). Preparation and characterization of free mixed-film of pectin/chitosan/Eudragit RS intended for sigmoidal drug delivery. Eur. J. Pharm. Biopharm. Off. J. Arb. Pharm. Verfahr..

[39] Školáková T., Slámová M., Školáková A., Kadeřábková A., Patera J., Zámostný P. (2019). Investigation of Dissolution Mechanism and Release Kinetics of Poorly Water-Soluble Tadalafil from Amorphous Solid Dispersions Prepared by Various Methods. Pharmaceutics.

[40] Liu H., Du K., Li D., Du Y., Xi J., Xu Y., Shen Y., Jiang T., Webster T.J. (2018). A high bioavailability and sustained-release nano-delivery system for nintedanib based on electrospray technology. Int. J. Nanomed..

[41] Nihei T., Ushiro E., Sato H., Onoue S. (2021). Biopharmaceutical Study on Nobiletin-Loaded Amorphous Solid Dispersion with Improved Hypouricemic Effect. Molecules.

[42] Jin S., Lee C.H., Lim D.Y., Lee J., Park S.-J., Song I.-S., Choi M.-K. (2021). Improved Hygroscopicity and Bioavailability of Solid Dispersion of Red Ginseng Extract with Silicon Dioxide. Pharmaceutics.

[43] Alshehri S., Alanazi A., Elzayat E.M., Altamimi M.A., Imam S.S., Hussain A., Alqahtani F., Shakeel F. (2021). Formulation, In Vitro and In Vivo Evaluation of Gefitinib Solid Dispersions Prepared Using Different Techniques. Processes.

